# NMR spectroscopy and chemometrics as a tool for anti-TNFα activity screening in crude extracts of grapes and other berries

**DOI:** 10.1007/s11306-012-0406-8

**Published:** 2012-02-17

**Authors:** Kashif Ali, Muzamal Iqbal, Henrie A. A. J. Korthout, Federica Maltese, Ana Margarida Fortes, Maria Salomé Pais, Robert Verpoorte, Young Hae Choi

**Affiliations:** 1 Natural Products Laboratory, Institute of Biology, Leiden University, 2300 RA Leiden, The Netherlands; 2 Fytagoras BV Plant Science, Sylviusweg 72, 2333 BE Leiden, The Netherlands; 3 Plant Systems Biology Lab, ICAT, Center for Biodiversity, Functional and Integrative Genomics, FCUL, 1749-016 Lisbon, Portugal

**Keywords:** Grapes, Developmental stages, NMR spectroscopy, Chemometrics, Anti-TNFα activity, PLS modeling

## Abstract

The identification of active ingredients in crude plant extracts offers great advantages. In this study, nuclear magnetic resonance and chemometrics were used for the screening of in vitro anti-TNFα activity in different berry types. Solid phase extraction was applied and the resulting water, methanol–water (1:1), and methanol fractions were tested for the activity. The methanol–water fraction contained most of the phenolics and showed significantly higher activity than the other two fractions. In the second phase of this study, grapes from ‘Trincadeira’, ‘Touriga Nacional’, and ‘Aragonês’, at four developmental stages were metabolically classified and tested for the TNFα inhibition. The initial stages of grape development, green and veraison, were found more active against TNFα production as compared to the later ripe and harvest stages. Among the cultivars, ‘Touriga Nacional’ was found to be the most potent inhibitor. Different multivariate data analyses algorithms based on projections to latent structures were applied to correlate the NMR and TNFα inhibition data. The variable importance in the projections plot showed that phenolics like quercetin, myricetin, (+)-catechin, (−)-epicatechin, caftarate, and coutarate, were positively correlated with high activity. This work demonstrates the great potential of NMR spectroscopy in combination with chemometrics for the screening of large set of crude extracts, to study the effects of different variables on the activity, and identifying active compounds in complex mixtures like plant extracts.

**Electronic supplementary material:**

The online version of this article (doi:10.1007/s11306-012-0406-8) contains supplementary material, which is available to authorized users.

## Introduction

Inflammation is a complex process and various mediators, like interleukins and tumor necrosis factor-α (TNFα), are involved in the development of inflammatory diseases. TNFα, an inflammatory mediator, is one of the most important pro-inflammatory cytokines. It was discovered in 1975 as having an anti-tumor activity, but is now recognized as a host defense factor in immunological and inflammatory responses (Tracey et al. 1994). TNFα is known to be secreted during early stages of acute and chronic inflammatory diseases such as rheumatoid arthritis, asthma, septic shock and other allergic diseases (Herath et al. 2003; Cho et al. 2001). Low production of TNFα is advantageous for the host but its overproduction during infection plays a pivotal role in the development of diseases like disseminated intravascular coagulation, death in septic shock, cerebral malaria, along with wide range of other inflammatory diseases including asthma, dermatitis, multiple sclerosis, inflammatory bowl disease, cystic fibrosis, rheumatoid arthritis, and immunological disorders (Björnsdottir and Cypcar 1999; Murphy et al. 1998; Medana et al. 1997). Therefore it is evident that the suppression of TNFα or anti-TNFα therapy could be beneficial for the treatment of these acute and chronic diseases.

Plants may serve as an interesting source for discovering new compounds that can be used in anti-TNFα therapy. Chemical phenotyping of plants has become the focal point in recent years, as the analysis of the low molecular weight compounds reflect the physiological activities of an organism or tissue under certain conditions. The observable chemical profile or fingerprint is highly complex consisting of a variety of compounds of very different nature. Considering the great chemical diversity, it is unlikely that a single analytical method could provide information about all the metabolites, and at the same time be unbiased, rapid, reproducible, and stable over time, while requiring only simple sample preparation.

An accurate snap shot of the metabolome is highly important in metabolomics, which requires a reliable metabolite extraction (Colquhoun 2007; Becknort et al. 2007). Many platforms are now available for the high throughput analysis of metabolites, varying in their sensitivity (Kopka et al. 2004). In case of a pure organic compounds, two of the most widely used parameters for solvent selection are total solubility and constituent partial solubility, but in metabolomics extraction is a totally different state of affairs. Based on sample chemistry and aim of the research, many extraction protocols for metabolomics studies have been published, offering different advantages but also having some limitations (Lisec et al. 2006; De Vos et al. 2007; Kruger et al. 2008; Kim et al. 2010). Solid phase extraction (SPE) has been an effective sample handling technique with advantages like high recovery, high pre-concentration factors, low organic solvent consumption, simplicity, and ease of operation (Zhao et al. 2007) and has been successfully used in many studies (Fraccaroli et al. 2008; Zou et al. 2007).

NMR has a unique place not only in structure elucidation, and characterization of molecules but is now also considered as a major tool in metabolomics studies. Though criticized because of its low sensitivity, NMR is known for advantages like non-destructive nature, easy sample preparation, and a relatively short analysis time. These and two other striking features of NMR, its non-selectivity and the use of NMR data directly for quantification, makes NMR an optimum choice for broad range metabolite analysis and quantification (Son et al. 2009; Dixon et al. 2006). NMR is now widely used in combination with different multivariate data analyses methods to do metabolic profiling of various samples (Brescia et al. 2002; Charlton et al. 2002). Characterization of different plant species (Kim et al. 2005), and cultivars (Ali et al. 2009), monitoring grape berry growth (Ali et al. 2011a), and the effects of growing areas, vintage, soil, and microclimate have been reported using the same combination (Pereira et al. 2005, 2006a). Many reports have been published on correlating the NMR and bioactivity data using various multivariate data analysis methods (Cho et al. 2009; Cardoso-Taketa et al. 2008).

Food items like fruits, spices, and herbs are well known for their anti-inflammatory properties (Mueller et al. 2010; Yuliana et al. 2011). Among the fruits, the use of grapes for multiple purposes like juice, fresh and dried fruit, and most importantly in wine production, make them one of the most economically important and widely cultivated fruit crops across the world. In addition to their economic importance, an increasing number of medicinal advantages have been attributed to grapes. Grapes phytochemistry is known to have relatively high concentrations of phenolics which in turn resulted in many health effecting properties, for instance, cardioprotective, anti-oxidant, anti-inflammatory, and anti-cancer activities (Ali et al. 2010). Studies using human (Zern et al. 2005), and animal (Fuhrman et al. 2005) models have shown that due to the abundance of polyphenols possessing anti-oxidative and anti-inflammatory properties, dried grape powder has cardioprotective effects.

The present study first describes the screening of different types of berries for the in vitro anti-TNFα assay in combination with SPE. As the second step, three Portuguese grape varieties at different development stages are analyzed for the same activity. Two different vintages of ‘Trincadeira’ cultivar are also compared. Several primary and secondary metabolites (especially phenolics) using 1D and 2D NMR techniques are identified. The correlation of activity and NMR data using different multivariate data analyses methods in order to identify the active ingredients in grapes and other berries is also presented.

## Materials and methods

### Sampling

Different types of berries, i.e. cranberry, blueberry, redberry, strawberry, raspberry, blackberry, and grapes (green, red, and black), were purchased from local markets in Netherlands and used for general screening. For each berry type five biological replicates were used for anti-TNFα assay. After the general screening, three elite Portuguese grapes cultivars i.e. ‘Trincadeira’, ‘Touriga Nacional’, and ‘Aragonês’, were used in this study. Five biological replicates of each cultivar of 80–100 berries from 8 to 10 plants were collected in 2008 and 2007 (for ‘Trincadeira’ only) corresponding to the developmental stages of EL 32 (green), 35 (veraison), 36 (ripe), 38 (harvest). EL refers to the modified Eichhorn and Lorenz developmental scale as described by Coombe (1995). Each biological replicate contained berries from a single row of plants. Four rows distant 3–10 m from each other were used for each variety. Plants from the three varieties were growing in the vineyard 15–30 m apart. Seeds were removed from all the berries prior to extraction.

### SPE

A sample of 100 mg of lyophilized berries was extracted with 2 mL of the mixture of water and methanol (2:8), with ultrasonication for 20 min at 25°C. The suspension was then centrifuged at 3,500 rpm and the supernatant was transferred to a round-bottom flask. The same procedure was repeated two more times and the supernatants were pooled together in the flask and taken to dryness with a rotary evaporator. This extract was subjected to SPE on SPE-C18 cartridges (Waters, Milford, MA, USA). Prior to its use, the SPE cartridge was prepared by elution of 10 mL of methanol followed by 10 mL of water. Then, the redissolved extract (1 mL of deionized water) was applied to the cartridge and eluted successively with 5 mL of water and then 5 mL of methanol:water (1:1) and finally with 5 mL of methanol. All three fractions were collected in round bottomed flasks and evaporated under vacuum and were used for further NMR analysis. All the solvents were purchased from Biosolve B.V. (Valkenswaard, the Netherlands).

### Growth of cells and lipopolysaccharides stimulation

Human monocyte-like histiocytic lymphoma cells U937 obtained from the ATCC (CRL-1593.2) were grown in RPMI-1640 medium, supplemented with 10% (v/v) fetal calf serum and 2 mM l-glutamine (Life technologies, Breda, The Netherlands) at 37°C, 5% CO_2_ in a humidified atmosphere. U937 monocytic cells (5 × 10^5^ cells per well) were plated in 96-well culture plate and then differentiated into macrophages using phorbol 12-myristate 13-acetate (PMA, 10 ng mL^−1^, overnight, Omnilabo, Breda, The Netherlands). The PMA-differentiated macrophages were allowed to recover from PMA treatment for 48 h, during which the culture medium was replaced daily. Lipopolysaccharides stimulation of cells was performed as described by Sajjadi et al. (1996).

### Cells treatment with plant extracts

Immediately after stimulation cells were treated with plant extracts (redissolved in DMSO) at the concentration of 100 μg mL^−1^ and then incubated at 37°C for 4 h. Supernatant were then collected and measured for TNFα content using the Human TNFα enzyme linked immunosorbent assay (ELISA) kit (R&D systems, Europe Ltd).

### Enzyme-linked immunosorbent assay for TNFα

TNFα in culture supernatants were determined by quantitative ‘sandwich’ enzyme-linked immunosorbent assay using paired antibodies purchased from (Biosource International, Inc.,USA). In brief, all wells of high-binding Immulon-plates (96 well NUNC MaxiSorp microplates) were coated with 100 μL of the capture antibody (anti-Human TNFα) (0.250 mg 0.125 mL^−1^). After overnight incubation at 4°C, plates were washed with washing buffer and blocked for 1 h with 1% bovine serum albumin in phosphate-buffered saline. Plates were aspirated and inverted on absorbent paper to remove excess liquid. Samples and standards were diluted with assay buffer. 100 μL of diluted standards (recombinant Human TNFα protein) were filled in 16 wells of first two columns of plates. Rests of the wells were filled with 100 μL of samples in 100 μg mL^−1^ concentration. Only DMSO was used as a control. Immediately 50 μL of working detection antibody (0.025 mg 0.125 mL^−1^) was plated in every well and then incubated for 2 h at room temperature with continuous shaking (700 rpm). The wells were washed again five times with washing buffer before addition of 100 μL of streptavidin-HRP to the wells and incubated at room temperature further for 30 min with continuous shaking at 700 rpm. Again wells were aspirated and washed five times before addition of 100 μL of TMB substrate and then incubated for 30 min at room temperature with continuous shaking (700 rpm). After 30 min the reactions were terminated by addition of 100 μL of 2 M H_2_SO_4_, and absorbance was determined using a microtiter plate reader (Bio-Tek Instruments Inc., Winooski, VT, USA) at 450 nm. The concentration of TNFα in the samples was calculated by comparison of the absorbance of the samples to the standard curve. The ratio (%) of TNFα inhibition was calculated by the equation, i.e. Inhibition (%) = 100 × (1 − *T*/*C*), where *T* represents the concentration of TNFα with grape extract while *C* was the concentration of TNFα with only DMSO.

### Cell viability assay

Cell viability after treatment with different plant extracts was determined by using MTT assay. Briefly, U937 cells having concentration of (5 × 10^5^ cells mL^−1^) were placed in a 96 wells plate. The culture media also contains extracts of different berries (100 μg mL^−1^) in the presence or absence of 200 μg mL^−1^ LPS at 37°C. After 2.5 h of incubation at 37°C, the medium was discarded and the formazan blue, which formed by reacting MTT with mitochondrial dehydrogenase in the living cells, was dissolved with 100 μL DMSO. The optical density (OD) was measured at 540 nm. The background signal inherent to the plates when no cell was present was subtracted from the absorbance obtained from each sample.

### NMR spectroscopy

The three fractions eluted from SPE were redissolved in 1 mL of methanol-*d*_4_. An aliquot of 800 μL of sample was transferred to the 5-mm NMR tube and used for the NMR analysis. The deuterated methanol was purchased from Cambridge Isotope Laboratories, Inc., Andover, MA, USA. ^1^H NMR spectra were recorded at 25°C on a 500 MHz Bruker DMX-500 spectrometer (Bruker, Karlsruhe, Germany) operating at a proton NMR frequency of 500.13 MHz. MeOH-*d*_4_ was used as the internal lock. Each ^1^H NMR spectrum consisted of 128 scans requiring 10 min and 26 s acquisition time with the following parameters: 0.16 Hz/point, pulse width (PW) = 30° (11.3 μs), and relaxation delay (RD) = 1.5 s. A pre-saturation sequence was used to suppress the residual H_2_O signal with low power selective irradiation at the H_2_O frequency during the recycle delay. FIDs were Fourier transformed with LB = 0.3 Hz. The resulting spectra were manually phased and baseline corrected, and calibrated to MeOH-*d*_4_ at 3.3 ppm, using XWIN NMR (version 3.5, Bruker). 2D NMR techniques were performed on a 600 MHz Bruker DMX-600 spectrometer (Bruker, Karlsruhe, Germany) operating at a proton NMR frequency of 600.13 MHz. *J*-resolved NMR spectra were acquired using 8 scans per 128 increments for F1 and 8 k for F2 using spectral widths of 5,000 Hz in F2 (chemical shift axis) and 66 Hz in F1 (spin–spin coupling constant axis). A 1.5 s relaxation delay was employed, giving a total acquisition time of 56 min. Datasets were zero-filled to 512 points in F1 and both dimensions were multiplied by sine-bell functions (SSB = 0) prior to double complex FT. *J*-resolved spectra tilted by 45°, were symmetrized about F1, and then calibrated, using XWIN NMR (version 3.5, Bruker). ^1^H–^1^H correlated spectroscopy (COSY) and heteronuclear multiple bonds coherence (HMBC) spectra were also recorded on a 600 MHz Bruker DMX-600 spectrometer (Bruker). The COSY spectra were acquired with 1.0 s relaxation delay, 6,361 Hz spectral width in both dimensions. Window function for COSY spectra was sine-bell (SSB = 0). The HSQC spectra were obtained with 1.0 s relaxation delay, 6361 Hz spectral width in F2 and 27,164 Hz in F1. Qsine (SSB = 2.0) was used for the window function of the HSQC. The HMBC spectra were recorded with the same parameters as the HSQC spectra except for 30,183 Hz of spectral width in F2. The optimized coupling constants for HSQC and HMBC were 145 Hz and 8 Hz, respectively.

### Data analysis and statistics

The ^1^H NMR spectra (from all SPE fractions) were automatically reduced to ASCII files. Spectral intensities were scaled to methanol signal (δ 3.30) and reduced to integrated regions of equal width (δ 0.04) corresponding to the region of δ 0.0–10.0. The regions of δ 4.85–4.95 and δ 3.2–3.4 were excluded from the analysis because of the residual signal of D_2_O and CD_3_OD, respectively. Bucketing was performed by AMIX software (Bruker) with scaling on total intensity. Principal component analysis (PCA) with scaling based on Pareto, while projections to latent structures (PLS), PLS-discriminant analysis (PLS-DA), bidirectional orthogonal PLS (O2PLS), and O2PLS-discriminant analysis (O2PLS-DA), with scaling based on Unit Variance were performed with the SIMCA-P + software (v. 12.0, Umetrics, Umeå, Sweden). The TNFα content was arbitrarily set as 100 in the positive control and all the other values are normalized to this (% activity) and shown in results. Means and standard deviations were calculated and means comparisons were made with ANOVA at a significance level <0.01.

## Results and discussion

### ^1^H NMR spectra visualization

For convenience ^1^H NMR spectrum can be roughly divided into three distinct regions. For amino acids and organic acids, resonances can be observed in the region of δ 0.80–4.00. Area from δ 4.00 to 5.50 is known as carbohydrate region while the remaining δ 5.50–8.50 region is considered as phenolic region. The ^1^H NMR spectra of three SPE fractions of ‘Trincadeira’ cultivar are shown in Online Resource Fig. S1 (A and B). It is evident from the figure that the three SPE fractions are quite different from each other in terms of contained metabolites. The water fraction shows mostly sugars and organic acids while the methanol fraction shows mostly amino acids and fatty acids with some resonance in phenolic region. The methanol:water fraction shows the presence of maximum amount of phenolics with relatively few sugars and amino acids.

The phenolic regions of ^1^H NMR spectra from all three grape cultivars are also shown and among the different grape cultivars, ‘Touriga Nacional’ is found to have highest phenolic content. It can also be observed that, in general, each developmental stage has a unique metabolic profile. As shown by NMR, the initial stage in berry growth is characterized by high levels of phenolics with fewer sugars and organic acids. As the berry grows, the level of sugars and organic acids seems to increase with a decrease in phenolics content. The distribution of metabolites according to grape cultivars and developmental stages is explained in detail in the later sections. The phenolic regions of ^1^H NMR spectra from the 2007 and 2008 vintages of ‘Trincadeira’ cultivar are also compared and shown in Online Resource Fig. S2. The figure clearly suggests the higher accumulation of phenolics in the harvest stage of 2007 vintage of the ‘Trincadeira’ variety.

### Metabolite identification

As a powerful analytical tool, ^1^H NMR offers many advantages in metabolomics studies but signals congestion in NMR spectra hampered the metabolite identification. Several 2D NMR techniques, like *J*-resolved, ^1^H-^1^H COSY, ^1^H-^13^C HMBC, and ^1^H-^13^C HSQC, provide additional information which facilitates the identification of metabolites. Among the above-mentioned techniques *J*-resolved and ^1^H-^1^H COSY are widely used due to short measuring time with good quantitative features. They showed to be quite effective in the confirmation of metabolites like phenylpropanoids and flavonoids (Viant 2003; Liang et al. 2006). Recently the potential of ^1^H-^13^C-related NMR techniques, like ^1^H-^13^C HMBC and ^1^H-^13^C HSQC, in metabolomics has been discussed (Hyberts et al. 2007; Lewis et al. 2007). The use of these two techniques is not yet very common in metabolomics because of the long measuring time.

Using our in-house library of NMR data of common metabolites, we identified some flavonoids including both flavonols and flavan-3-ols. Flavonols like quercetin and myricetin were identified in the aromatic region along with (+)-catechin and (−)-epicatechin of the flavan-3-ols group. The aromatic part of the ^1^H NMR spectra shows some signals of hydroxybenzoates like gallic acid, syringic acid, and vanillic acid. Tartaric esters of hydroxycinnamic acid were also identified which include caftaric acid (caffeic acid conjugated with tartaric acid), fertaric acid (ferulic acid conjugated with tartaric acid), and *p*-coutaric acid (coumaric acid conjugated with tartaric acid). Along with the *trans*-forms, the *cis*-forms of these conjugated cinnamic acids, i.e. *cis*-caftaric acid and *cis*-coutaric acid, were also detected.

The flavonoids quercetin and myricetin were identified in the aromatic region. The quercetin signal at *δ* 6.49 of H-8 correlates in the ^1^H-^1^H COSY spectrum with the signal at *δ* 6.27 of H-6 and a signal at *δ* 6.95 of H-5′ with one at *δ* 7.56 of H-6′. Similar correlations for the signals of myricetin at δ 6.51 of H-8 with δ 6.29 of H-6 are also present in the ^1^H-^1^H COSY. Resonances of H-8′ and H-7′ (olefinic protons) of *trans*-hydroxycinnamic acids are clearly observed as doublets of 16.0 Hz in the range of δ 6.39–6.50 and δ 7.59–7.70, respectively, in *J*-resolved spectrum. These protons are also found to correlate in the ^1^H-^1^H COSY spectra, and continued by the coupling with a carbonyl carbon at δ 168.3 in the HMBC spectra. These signals are assigned to cinnamic acids derivatives including caffeic acid, *p*-coumaric acid, and ferulic acid. In the ^1^H NMR spectra of grape berry samples, these resonances were assigned to three different hydroxycinnamic acids moieties which include *trans*-caffeoyl, *trans*-coumaroyl, and *trans*-feruloyl derivatives. The ^1^H-^1^H COSY spectra show correlations among signals like δ 6.41 with δ 7.62, and δ 7.02 with δ 6.88 of caffeoyl; δ 7.51 with δ 6.87, and δ 6.45 with δ 7.65 of coumaroyl; δ 6.46 with δ 7.56 of feruloyl derivative.

These cinnamic acids were also found to be conjugated with tartaric acid via an ester linkage. The signal for tartaric acid was observed in the region of δ 5.32–5.44 in the ^1^H NMR spectrum, being shifted downfield from the typical tartaric acid signal at δ 4.30 due to the bonding to the carboxylic function of cinnamic acids which was confirmed by their correlation with the signals in the region of δ 167.5–168.5 in the HMBC spectra. Based on these assignments, these compounds were identified as *trans*-caftaric acid (caffeic acid conjugated with tartaric acid), *trans*-fertaric acid (ferulic acid conjugated with tartaric acid), and *trans*-*p*-coutaric acid (coumaric acid conjugated with tartaric acid).

Along with the *trans*-forms, the *cis*-forms of these cinnamic acids, i.e. *cis*-caftaric and *cis*-*p*-coutaric acid, were also detected. When compared to their *trans*-configuration, the *cis*-forms showed an upfield shift of the signals for H-8′ and H-7′ along with the reduction in the coupling constant from 16.0 to 13.0 Hz. Two clear doublets of 13.0 Hz at δ 5.92 and δ 5.94 were detected for the H-8′ in the *cis*-configuration. The ^1^H-^1^H COSY spectra also confirmed this by showing the correlation of these signals with the respective H-7′ protons at δ 6.81 and δ 6.86. It was also confirmed by the correlation of this signal with the carbonyl resonance at δ 167.2 in the HMBC spectra.

A number of amino and organic acids were identified due to the high signal intensities in the amino acid region. Amino acids like alanine, leucine, threonine, valine, proline, methionine, and glutamate, were identified by comparison with the reference spectra of these compounds. The signals in the carbohydrate regions were highly clustered and overlapped. This region showed the signals of the anomeric protons of β-glucose, α-glucose, fructose, and sucrose. Resonances for some other compounds like GABA, choline, and 2,3-butanediol were also identified in the same region. A number of signals have been elucidated as organic acids like α-linolenic acid, acetic acid, succinic acid, fumaric acid, formic acid, citric acid, malic acid, and tartaric acid. All these assignment of metabolites are based on previous studies and our in house data base NMR data of standards measured under identical conditions and our previous reports (Ali et al. 2009, Ali et al. 2011a, b; Abdel-Farid et al. 2007; Liang et al. 2006).

### Anti-TNFα activity screening in berries

As the first step in this study, all three fractions of SPE from different berries extracts are tested for in vitro inhibition of TNFα production in LPS stimulated U937 cells (Online Resource Fig. S3). The water and methanol fractions were found having the least TNFα inhibitory activity with no significant difference among different berry types. The methanol:water fraction shows maximum TNFα inhibition with cranberry show significantly higher activity than all the other berry types except black grapes. Among the red, white, and black grapes, it is interesting to note that black and white grapes are with significantly higher activities than red grapes. Among the other berry type, raspberry and blueberry show significantly higher activities than the redberry and strawberry.

### Multivariate data analysis for berries

Multivariate data analysis algorithms are an essential component of any metabolomics studies. These methods are used to reduce the dimensionality of multivariate dataset and thus enable to recognize possible differences or similarities among the samples. PCA is considered as a primary tool in metabolomics used to reduce the dimensionality of a multivariate dataset, and thus helping to better understand possible differences between classes. It is an unsupervised method hence the clustering or separation of samples is purely due to similarities or differences, respectively, among all the samples. The NMR data from the SPE fractions of all the samples were subjected to PCA in order to highlight the differences between the SPE fractions and to identify the metabolites responsible for that distinction.

The PCA score plot shows good separation among the SPE fractions (Online Resource Fig. S4). The methanol fractions are totally separated from the other two fractions by component 1 (56.6%) while the water and methanol:water fractions are separated by component 2 (8.8%). By examining the loadings plot and the respective NMR spectra, it is clear that all the three fractions are quite different in their metabolic contents. The methanol fraction is found to be higher in fatty acids with very small quantities of phenolics and amino acids. The water fraction is relatively higher in sugars and some amino acids and organic acids. Most of the phenolics are found to elute in the methanol:water fraction.

**Fig. 1:**
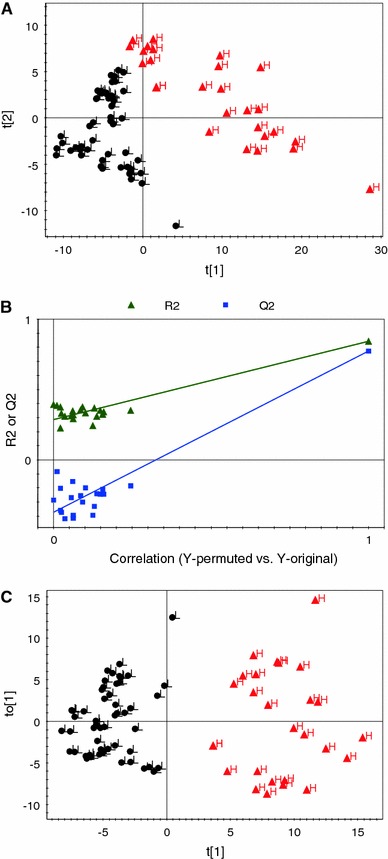
The PLS score plot (**a**), permutation test for PLS (**b**), and score plot of O2PLS (**c**) for general screening in different berry types are shown. Samples with *black color* are with low while samples with *red color* are with high inhibitory activity against TNFα production (Color figure online)

In order to identify the metabolites responsible for in vitro inhibition of TNFα production, a supervised method, i.e. PLS, is used. It is a supervised method in which the actual data from the bioactivity assay are used as a *Y*-variable. The PLS analysis was found effective in separating the high and low activity samples (Fig. 1a). The application of O2PLS resulted in much better distinction of the samples with different activities than the PLS-DA model (Fig. 1c). By examining the corresponding loadings plot of O2PLS-DA, metabolites responsible for the separation of high activity samples from the low activity samples are identified. Among the phenolics, gallic acids, quercetin, myricetin, (+)-catechin, caffeic acid, and ferulic acid were found discriminating between the classes and suggests their involvement in the inhibition of TNFα production.

One of the key aspects of a supervised regression algorithm is model validation. A permutation test is often used for validation of methods like PLS. A permutation test is the calculation of goodness of fit and the predictive ability of the model, R2 and Q2, respectively. The R2 value can vary from 0 to 1, where 1 means a model with a perfect fit. If the Q2 value is more than 0.5, the model is considered to have good predictability and if it is higher than 0.9 and less than 1.0, then the model is considered to have an excellent predictability. It is suggested that if more than five PLS components are included in the model the training set data generally reproduce excellently. The R2 and Q2 values of PLS was calculated using four components. For the inhibitory activity against TNFα production the R2 and Q2 values for PLS analysis were 0.84 and 0.77, respectively. This PLS model was validated by the permutation method through 20 applications in which all Q2 values of permuted Y vectors were lower than original ones and the regression of Q2 lines intersect at below zero (Fig. 1b).

### Grapes and anti-TNFα activity

The next part of the study is to analyze the potential of three grape cultivars at four developmental stages to inhibit the production of TNFα is evaluated. All three fractions from SPE of grape extracts were tested for anti-TNFα activity at 100 μg mL^−1^. The methanol:water fractions show significantly higher activity than the water and methanol fractions. It has been shown in the previous section that the metabolic composition of these fractions are quite different from each other and the methanol:water fraction contained most of the grape phenolics. The water and methanol, fractions also showed some activity, though mostly not significantly different from each other.

All the methanol:water extracts of the three cultivars show variable activity at different developmental stages (Online Resource Fig. S5). The veraison stage is found to have maximum anti-TNFα activity in every cultivar followed by the green stage. For two cultivars, ‘Touriga Nacional’ and ‘Aragonês’, the ripe and harvest stages are not significantly different in anti-TNFα activity. The ripe stage of ‘Trincadeira’ is significantly higher than the harvest stage in inhibiting the TNFα production. Among the green stages of all three cultivars, the green ‘Touriga Nacional’ grapes are found more active than the green grapes of ‘Trincadeira’ and ‘Aragonês’ (Online Resource Fig. S6). At veraison, the ‘Aragonês’ and ‘Touriga Nacional’ grapes are not different but both are significantly more active than ‘Trincadeira’. All the three cultivars show similar potency of inhibiting TNFα production at the ripe and harvest stages.

In order to highlight the vintage effect on anti-TNFα activity, the 2007 and 2008 vintage of ‘Trincadeira’ cultivar are compared. ‘Trincadeira’ 2007 shows highest anti-TNFα activity at veraison stage followed by green stage but unlike ‘Trincadeira’ 2008 (see above), the 2007 vintage shows no significant difference in TNFα inhibition at later stages of development i.e. ripe and harvest (Online Resource Fig. S5). Comparing every developmental stage of these two vintages, only green and harvest are different. Green ‘Trincadeira’ 2008 grapes show higher activity while at harvest ‘Trincadeira’ 2007 grapes show significant inhibition of TNFα production. The ^1^H NMR spectra analysis shows that ‘Trincadeira’ 2007 has more phenolics at harvest as compared to ‘Trincadeira’ 2008, this suggesting a relationship between phenolics and activity (Online Resource Fig. S2, S7).

### Grapes cultivars, development stages, and SPE fractions differentiation

**Fig. 2:**
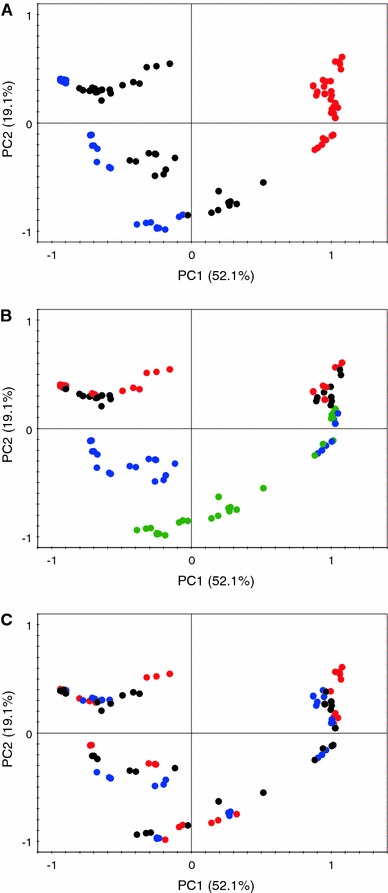
The principal component analysis score plots. In **a**, samples are colored according to SPE fractions where *red* represents methanol, *black* represents methanol:water (1:1), and *blue* represents water fraction. In **b**, samples are colored according to developmental stages where *green* represents *green* stage, *blue* represents veraison, *black* represent ripe, and *red* represents harvest stage. In **c**, samples are colored according to grape cultivars where *red* represents samples from Aragonês, *blue* represents samples from ‘Touriga Nacional’, and *black* represents samples from ‘Trincadeira’ (Color figure online)

The NMR data from the SPE fractions of all the samples have been subjected to PCA in order to identify possible markers for the different cultivars, developmental stages, and SPE fractions. Figure 2 shows the score plots of PCA where samples are colored according to SPE fractions, cultivars, and developmental stages. Figure 2a represents the PCA score plot where samples are colored according to SPE fractions. Similar to general screening of berries, all three fractions of SPE are well separated. The water fractions are clustered on the negative side of PC1 while the methanol fractions are grouped on the positive side of PC1. The methanol:water fractions are located in between the methanol and water fractions, mostly having negative PC1 values. The SPE fractions are, like in the case of general berries screening, very much distinct in their metabolic contents. The water fraction is relatively higher in sugars and some amino acids and organic acids. Most of the phenolics are found to elute in the methanol:water fraction while the remaining amino acid and phenolics come out with the last methanol fraction. The methanol fraction is also found higher in fatty acids.

To highlight the differences based on developmental stages, samples from the same PCA are colored according to developmental stages in Fig. 2b. It is obvious from the score plot that while PC1 is responsible for the separation of SPE fractions, PC2 (19%) is quite effective in discriminating the developmental stages of grapes. The initial stages, like green and veraison, are on the negative side of PC2 whereas the remaining stages, like ripe and harvest, mostly have positive PC2 scores. The same PCA score plot is shown in Fig. 2c but this time the samples are colored according to grape cultivar. It is evident from this figure that PCA is not very effective as the samples are not clustered based on the grape cultivars. The corresponding loading plots with the respective NMR spectra reveal the information regarding the metabolites responsible for the differentiation of samples on the score plots. The grapes in green and veraison stages have higher levels of phenolics with relatively less sugar and organic acid contents. As the berries grow the level of phenolics start to decrease whereas sugars and organic acids concentrations increase. A detailed account of the distribution of metabolites based on these cultivars at these development stages is given in our recent report (Ali et al. 2011a).

### Vintages, development stages, and SPE fractions differentiation

SPE fractions of grapes from 2007 and 2008 vintages of the ‘Trincadeira’ cultivar at four developmental stages were analyzed and compared for metabolic differences and TNFα inhibition. PCA, also in this case, is found effective in highlighting the metabolic differentiation among the samples based on developmental stages and SPE fractions and responsible metabolites are identified (Online Resource Fig. S8). As discussed above, phenolics are the main discrimination factor in SPE fractions while a similar metabolic behavior of developmental stages was observed in all grape cultivars. However, to analyze specifically the vintage effects on the grape metabolic profile, supervised multivariate data analysis was applied.

**Fig. 3:**
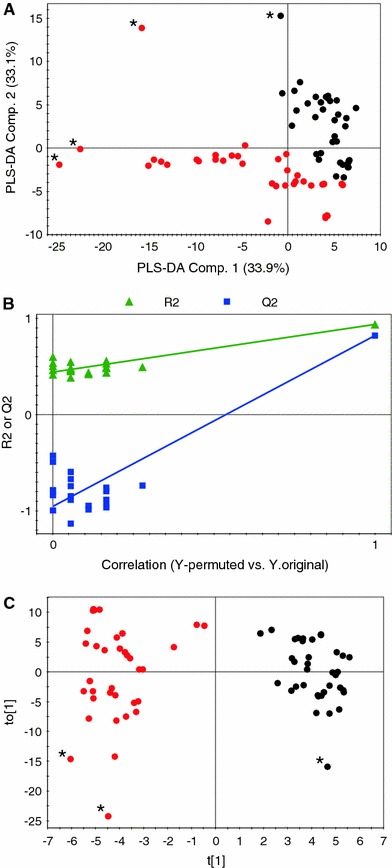
Multivariate data analysis of ‘Trincadeira’ 2007 and 2008. The PLS-DA score plot (**a**), permutation test for PLS-DA (**b**), and score plot of O2PLS-DA (**c**) are shown. Samples with *black color* are from ‘Trincadeira’ 2007 while samples with *red color* are from ‘Trincadeira’ 2008. Samples with *Asterisk* represents an outlier (Color figure online)

First, PLS-DA was used in which samples are classified into two classes based on samples from 2007 to 2008 vintages. The score plot (Fig. 3a) shows good separation among the samples belonging to the two different classes but none of the components is found totally effective. The PLS-DA model was validated using permutation test with 20 applications (Fig. 3b). To draw clear conclusions, O2PLS-DA was applied. The score plot (Fig. 3c) shows very clear distinction among the different vintages. The loading plot shows that the 2007 vintage has higher levels of phenolics than the 2008 vintage. The 2008 vintage shows elevated levels of organic acids like malate and citrate with some sugars like glucose and fructose.

### Multivariate data analysis for grape metabolome and activity correlation

**Fig. 4:**
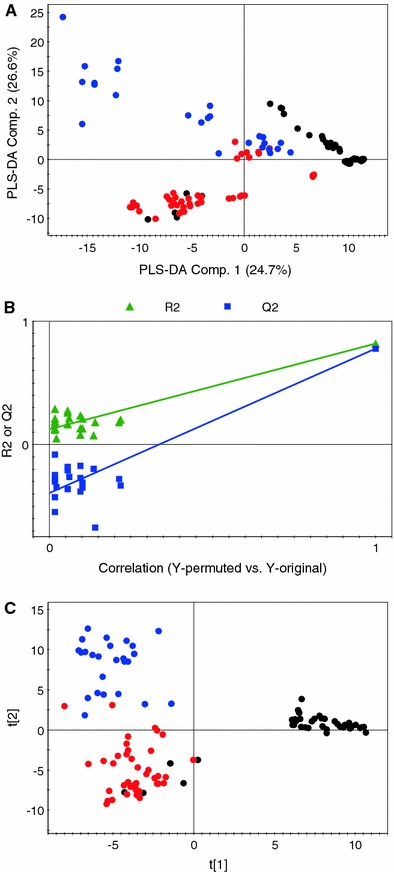
The score plots of PLS-DA (**a**) and O2PLS-DA (**c**) are represented. Samples with *black color* are of low anti-TNFα activity while samples with *red* and *blue colors* are of medium and high anti-TNFα activity, respectively. The permutation test for PLS-DA (**b**) is also presented (Color figure online)

In this study, based on activity data, we classify our samples into low (<10%), medium (≥10 and <25%), and high (≥25%) activity classes as *Y*-variables, and used these in a PLS-DA. Figure 4a shows that this gives a clear separation especially the samples with the lowest and medium activity are grouped separately. Samples with high activity are scattered and some are mixed with the samples with medium activity. This PLS-DA model was validated by a permutation test. The R2 and Q2 values of PLS-DA using 6 components were calculated. For anti-TNFα activity the figures were 0.82 and 0.78, respectively. This PLS-DA model was validated by the permutation method through 20 applications in which all Q2 values of permuted Y vectors were lower than original ones and the regression of Q2 lines intersect at below zero (Fig. 4b).

In order to get better separation, especially for the samples with high activity, O2PLS-DA is applied. The score plot of O2PLS-DA (Fig. 4c) shows much better separation among the samples based on anti-TNFα activity. Samples with low, medium, and high activity are nicely separated on the score plot. Few samples from low activity and medium activity classes and mixed with the medium activity and high activity classes, respectively, as their anti-TNFα activity values are on the border line of their classes. The O2PLS-DA model is validated by cross validation-analysis of variance (CV-ANOVA) with a *p*-value of 8.35 × 10^−38^. By examining the corresponding loadings plot, the metabolites responsible for separation are identified. Samples with different activity levels mainly differ in their phenolic contents. The high anti-TNFα activity samples have higher levels of phenolics like cinnamic acids, flavonols, and flavan-3-ols while the medium and low activity samples have less or no phenolic contents.

**Fig. 5:**
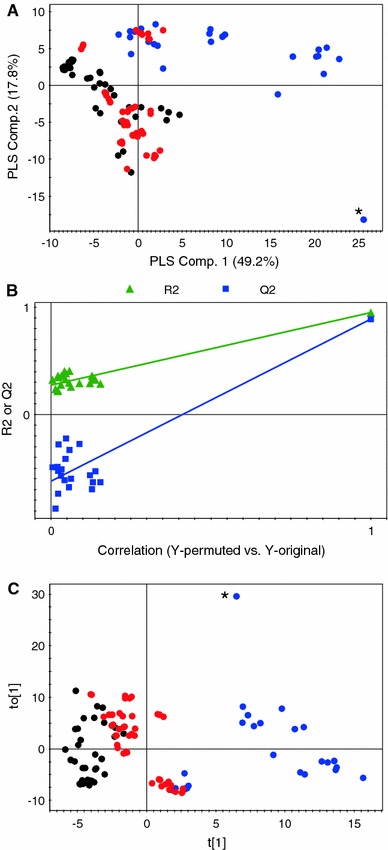
The score plots of PLS (**a**) and O2PLS (**c**) are represented. Samples with *black color* are of low anti-TNFα activity while samples with *red* and *blue colors* are of medium and high anti-TNFα activity, respectively. The permutation test for PLS (**b**) is also presented (Color figure online)

The next step is to perform the direct correlation between the activity and NMR data using original anti-TNFα assay values. Instead of classifying samples as high, medium, and low activity groups, the activity data from TNFα assay for each sample are used directly. In such approaches two different data sets, independent variable (like NMR spectral data) and dependent variable (like anti-TNFα activity), are correlated using regression. For this purpose, PLS analysis was performed using the NMR and activity data. The PLS score plot (Fig. 5a) shows relatively good separation among the samples but many are overlapped with the other groups. Component 1 is mainly responsible for the separation as the samples are arranged from low to high activity along the negative to positive side of component 1, respectively. For PLS modeling again the permutation method through 20 applications was used for validation. The regression of Q2 lines intersect at below zero with all Q2 values of permuted Y vectors were lower than original. Variance (R2) and cross-validated variance (predictive ability of the model, Q2) values of PLS using seven components were calculated and for anti-TNFα activity the figures were 0.95 and 0.89, respectively (Fig. 5b).

Finally for data correlation, we used another multivariate data analysis method known as bidirectional orthogonal PLS (O2PLS). Analyses like PLS regression can cause systematic variation of any data block due to structured noise present in the data blocks. Other algorithms, like O2PLS-DA and O2PLS, are multivariate projection methods which remove the structured noise by extracting linear relationships from independent and dependent data blocks, in a bidirectional way, and results in the decomposition of systematic variation into two model parts: the predictive or parallel part and the orthogonal part (Trygg and Wold 2002, 2003). The score plot, Fig. 5c, showed very nice separation among low, medium and high activity samples based on component 1. This O2PLS model was validated by CV-ANOVA with *p*-value of 1.4 × 10^−37^. Like PLS-DA and O2PLS-DA the corresponding loadings plot show that the samples with high anti-TNFα activity contained more phenolics, such as cinnamates and flavonoids, when compared to samples with low and medium activity.

In PLS based regression, VIP (variable importance in the projections) can be defined as a weighted sum of squares of the PLS weights. It has been indicated that it is directly proportional with the influence of factor on the separation on score plot, meaning, factors have higher VIP values are more important for the samples separation. For O2PLS-DA and O2PLS analyses, VIP values for several phenolic compounds, responsible for separation on the score plot, are presented in Table 1. As indicated, in O2PLS-DA analysis, caftaric acid, and (+)-catechin are the metabolites with top two VIP scores while quercetin and myricetin have relatively less VIP values. In O2PLS model, again (+)-catechin is one of the top two followed by coutaric acid while caftaric acid shown a much lower VIP score. This high VIP scores for the identified phenolics legitimate their involvement in the separation of high activity samples and suggest a role of these compounds in inhibiting TNFα production.

**Table 1:** The VIP (variable importance in the projections) values of the major contributing compounds for the separation in the score plots derived from O2PLS-DA and O2PLS models

Compounds	Chemical shift (ppm)^a^	VIP values	
		O2PLS-DA	O2PLS
Quercetin-3-*O*-glucoside	6.27 (d, *J* = 2.0, H-6)	1.38	2.06
Myricetin	6.51 (d, *J* = 2.0, H-8)	1.17	1.62
(+)-Catechin	6.75 (d, *J* = 8.0, H-5′)	1.44	2.16
(−)-Epicatechin	5.96 (d, *J* = 2.2, H-6)	1.40	2.09
Caftaric acid	7.62 (d, *J* = 16.0, H-7′)	1.55	1.80
Fertaric acid	6.32 (d, *J* = 16.0, H-8′)	1.43	2.08
*p*-Coutaric acid	7.65 (d, *J* = 16.0, H-7′)	1.41	2.10

^a^Chemical shifts of the metabolites are shown with the splitting, coupling constants (Hz), and the corresponding protons

The ^1^H NMR spectra clearly shows distinction among different SPE fractions of general berries extracts and specifically grape cultivars and vintages, and their developmental stages. This clearly advocates the enormous analytical potential of NMR spectroscopy as compared to other platforms for metabolomics studies (Verpoorte et al. 2008). Multivariate data analysis in combination with NMR is very popular in metabolic phenotyping studies of plants. Many reports have been published regarding grape berries using the same approach (Pereira et al. 2005, 2006a, b; Son et al. 2009). In this study, metabolic profiling of different grapes at different stages of ripening has been successfully performed. The initial stages, green and veraison, have been characterized with high phenolics, whereas high sugar and organic acids content is observed in the later stages i.e. ripe and harvest, as also reported previously (Ali et al. 2011). This metabolic distinction among the developmental stages reflect in the associated anti-TNFα activity as green and veraison are found more active than ripe and harvest.

The vintage effect on grape metabolome is quite obvious now as it is widely accepted that the several climatic factors are involved in the biosynthesis of several key metabolites in grapes (Pereira et al. 2006b). The green and harvest stages of these two vintages present significant metabolic differences, characterized by higher and lower phenolic contents in 2008 vintage, respectively, as compared to 2007 vintage. Since vintage has shown its effects on the phenolic contents of ‘Trincadeira’, the anti-TNFα activity shown by these vintages is also affected. The green and harvest stages from 2008 to 2007 vintages, respectively, showed significantly different anti-TNFα activity. As shown by the NMR spectra, this is due to difference in phenolic contents. It has been reported that different factors like hot and dry climate can result in higher phenolic contents in grapes (Pereira et al. 2006b). For instance, the insolation totals were higher in July and August of 2007 and differences in rain totals and average temperature were also observed in between seasons and may influence the fine tuning of phenolics’ biosynthesis (unpublished data). It is interesting to note that transcriptomic analysis using Affymetrix GrapeGen^®^ genome array showed that a gene coding for anthocyanidin reductase which is involved in proanthocyanidins biosynthesis such as catechin was more expressed in 2007 samples (Online Resource Table ST1). Since catechin seems to present high anti-TNFα activity as suggested by the results hereby presented this may constitute a good example of positive integration of transcriptomic and metabolomic data, and medicinal properties that deserves further attention.

Data correlation using different multivariate data analysis tools is now increasingly popular and found efficient in predicting the unknown NMR signals (metabolites) by using the resulting training model (Eriksson et al. 2006). Many reports have been published targeting to develop the predictive models for certain pharmacological activities in plants. Plants like *Hypericum perforatum* (Roos et al. 2004), *Artmesia annua* (Bailey et al. 2004), *Citrus grandis* (Cho et al. 2009), and *Galphimia glauca* (Cardoso-Taketa et al. 2008), have been efficiently studied for the prediction of different medicinal properties, using this approach. Such chemometrics based approach can provide first hand knowledge regarding the plant extracts and any related bioactivity without any tedious chromatographic separations.

Since grapes are one of the richest sources of polyphenolics, many studies (Chuang et al. 2010) have shown their potency against TNFα production as grape polyphenolics are widely acclaimed and accepted to have as anti-oxidative and anti-inflammatory properties (Baur et al. 2006; Breksa et al. 2010). Phenolics in grapes, like resveratrol (Stewart et al. 2008) and quercetin (Rivera et al. 2008) are known to reduce inflammation, while others like cinnamates, benzoates, flavonols, flavan-3-ols, and anthocyanins, are well known antioxidants (Lee et al. 2009). The present study is the only known attempt to analyze different grape cultivars, their developmental stages, and vintages for TNFα inhibition. The identified NMR signals, responsible for the activity, are related to quercetin, myricetin, (+)-catechin, (−)-epicatechin, coutaric acid, fertaric acid, and caftaric acid, which are found relatively higher in the samples with high activity using different chemometrics methods.

## Conclusion

Nuclear magnetic resonance spectroscopy has been applied for the phenotyping of three grape cultivars from Portugal at different development stages. SPE was used resulted in water, methanol:water (1:1), and methanol fractions and have been tested for TNFα inhibition. Using the presented approach, the analysis of NMR shifts in relation to pharmacological activity can provide information about what part of the NMR spectrum correlates with the activity and gives information about the active ingredients in crude extracts of medicinal plants. This approach proved to be an effective tool to short list a large set of crude extracts based on bioactivity. The effect of different variables on the activity of a sample can also be measured. Using this approach, compounds related to activity can be identified without extensive and elaborate chromatographic separation, and thus allows rapid identification of extracts with biological activity.

## Electronic supplementary material

Below is the link to the electronic supplementary material.

Supplementary material 1 (DOC 167 kb)

Supplementary material 2 (DOC 124 kb)

Supplementary material 3 (DOC 32 kb)

Supplementary material 4 (DOC 72 kb)

Supplementary material 5 (DOC 33 kb)

Supplementary material 6 (DOC 31 kb)

Supplementary material 7 (DOC 30 kb)

Supplementary material 8 (DOC 59 kb)

Supplementary material 9 (XLS 30 kb)
