# Supplementary material for: NMR spectroscopy and chemometrics as a tool for anti-TNFα activity screening in crude extracts of grapes and other berries
Source: Metabolomics. 2012 Feb 17;8(6):1148–61. doi: 10.1007/s11306-012-0406-8 (PMC3483097; doi:10.1007/s11306-012-0406-8)
Supplement: Supplementary file 1 — Supplementary material 1 (DOC 167 kb) [file 11306_2012_406_MOESM1_ESM.doc]

**Article title:** NMR spectroscopy and chemometrics as a tool for anti-TNFα activity screening in crude extracts of grapes and other berries

**Journal name:** Metabolomics

**Authors:** Kashif Ali^a^, Muzamal Iqbal^a^, Henrie A. A. J. Korthout^b^, Federica Maltese^a^, Ana Margarida Fortes^c^, Maria Salomé Pais^c^, Robert Verpoorte^a^, Young Hae Choi^a*^

**Affiliations:** ^a^ Natural Products Laboratory, Institute of Biology, Leiden University, 2300 RA Leiden, The Netherlands

^b^ Fytagoras BV Plant Science, Sylviusweg 72, 2333 BE Leiden, The Netherlands

^c^ Plant Systems Biology Lab, ICAT, Center for Biodiversity, Functional and Integrative Genomics, FCUL, 1749-016 Lisboa, Portugal

***Corresponding Author’s email:** [y.choi@chem.leidenuniv.nl](mailto:y.choi@chem.leidenuniv.nl)

**Fig. S1** Comparison of ^1^H NMR spectra of phenolic (A) and aliphatic (B) regions of SPE fractions (M=methanol, W=water, M:W=methanol:water) from the ‘Trincadeira’ cultivar, and phenolic region of three grape cultivars at green stage (C). All the spectra were recorded at 25 °C on a 500 MHz Bruker DMX-500 spectrometer (Bruker, Karlsruhe, Germany) operating at a proton NMR frequency of 500.13 MHz and scaled with the methanol-*d*_4_ (δ 3.3).
