# Supplementary material for: NMR spectroscopy and chemometrics as a tool for anti-TNFα activity screening in crude extracts of grapes and other berries
Source: Metabolomics. 2012 Feb 17;8(6):1148–61. doi: 10.1007/s11306-012-0406-8 (PMC3483097; doi:10.1007/s11306-012-0406-8)
Supplement: Supplementary file 6 — Supplementary material 6 (DOC 31 kb) [file 11306_2012_406_MOESM6_ESM.doc]

**Article title:** NMR spectroscopy and chemometrics as a tool for anti-TNFα activity screening in crude extracts of grapes and other berries

**Journal name:** Metabolomics

**Authors:** Kashif Ali^a^, Muzamal Iqbal^a^, Henrie A. A. J. Korthout^b^, Federica Maltese^a^, Ana Margarida Fortes^c^, Maria Salomé Pais^c^, Robert Verpoorte^a^, Young Hae Choi^a*^

**Affiliations:** ^a^ Natural Products Laboratory, Institute of Biology, Leiden University, 2300 RA Leiden, The Netherlands

^b^ Fytagoras BV Plant Science, Sylviusweg 72, 2333 BE Leiden, The Netherlands

^c^ Plant Systems Biology Lab, ICAT, Center for Biodiversity, Functional and Integrative Genomics, FCUL, 1749-016 Lisboa, Portugal

***Corresponding Author’s email:** [y.choi@chem.leidenuniv.nl](mailto:y.choi@chem.leidenuniv.nl)

**Fig. S6** TNFα inhibition ratio (%) exhibit by different grape cultivars at same developmental stages. Bars represent the Mean ± S.D. (*n*=3) at *p*<0.01
